# Factors associated with psychotropics adverse effects in elderly psychiatric inpatients

**DOI:** 10.1192/j.eurpsy.2024.1326

**Published:** 2024-08-27

**Authors:** W. Abid, M. Turki, B. Ben jmeaa, A. Zribi, M. A. Megdiche, S. Ellouze, N. Halouani, J. Aloulou

**Affiliations:** Psychiatry B department, Hedi chaker university hospital, sfax, Tunisia

## Abstract

**Introduction:**

Adverse effects (AEs) of psychotropic drugs are more frequent and potentially more dangerous in elderly subjects (ES), probably due to a greater frequency of somatic comorbidities, as well as polymedication.

**Objectives:**

The aims of this study were to determine the prevalence of AEs of psychotropic treatment among ES hospitalized in psychiatry, and to identify the associated sociodemographic and clinical factors.

**Methods:**

We conducted a retrospective and descriptive study. It concerned male patients aged at least 60 years, hospitalized in the psychiatry B department at CHU Hedi Chaker (Sfax, Tunisia) between 2018 and 2022. We collected demographic and clinical data from their medical records using a pre-established form.

**Results:**

We included 30 patients. The average age was 64 years. Addictive behaviors were reported in 60%, and somatic histories were noted in 53.3% of patients. The three most frequent psychiatric diagnoses were schizophrenia (43.3%), bipolar disorder (33.3%) and depressive disorder (13.3%). Among our patients, 10% experienced adverse psychotropic drug reactions: orthostatic hypotension 6.7%; neurological AEs 3.3%. Univariate analysis showed no significant relationship between sociodemographic variables and psychotropic drug AEs. Patients with bipolar disorder were more likely to develop AEs of psychotropic treatment (p=0.04).

**Conclusions:**

Our results suggest that special attention should be paid to avoiding psychotropic medication AEs in psychiatric inpatients ES. Indeed, extra precautions need to be taken in this population due to their reduced ability to report their symptoms.

**Disclosure of Interest:**

None Declared

